# Detergent-free Lipodisq Nanoparticles Facilitate High-Resolution Mass Spectrometry of Folded Integral Membrane Proteins

**DOI:** 10.1021/acs.nanolett.0c04911

**Published:** 2021-03-31

**Authors:** Kin Kuan Hoi, Juan Francisco Bada Juarez, Peter J. Judge, Hsin-Yung Yen, Di Wu, Javier Vinals, Garrick F. Taylor, Anthony Watts, Carol V. Robinson

**Affiliations:** † Department of Chemistry, Physical and Theoretical Chemistry Laboratory, University of Oxford, South Parks Road, Oxford OX1 3QZ, United Kingdom; ‡ Department of Biochemistry, Biomembrane Structure Unit, University of Oxford, Oxford OX1 3QU, United Kingdom; § OMass Therapeutics, The Schrödinger Building, Oxford Science Park, Oxford OX4 4GE, United Kingdom

**Keywords:** Native mass spectrometry, lipid−protein interactions, bacteriorhodopsin, Lipodisq, SMALP, post-translational modification

## Abstract

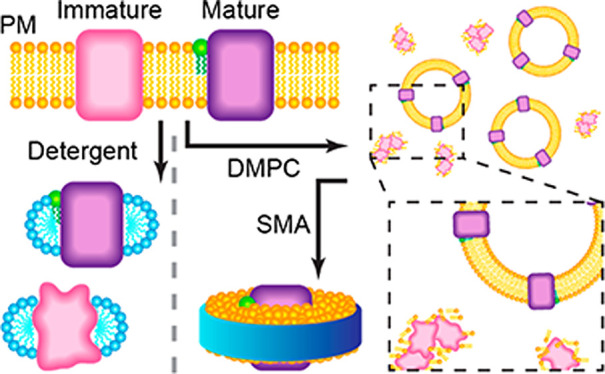

Integral membrane proteins pose considerable challenges to mass spectrometry (MS) owing to the complexity and diversity of the components in their native environment. Here, we use native MS to study the post-translational maturation of bacteriorhodopsin (bR) and archaerhodopsin-3 (AR3), using both octyl-glucoside detergent micelles and lipid-based nanoparticles. A lower collision energy was required to obtain well-resolved spectra for proteins in styrene-maleic acid copolymer (SMA) Lipodisqs than in membrane scaffold protein (MSP) Nanodiscs. By comparing spectra of membrane proteins prepared using the different membrane mimetics, we found that SMA may favor selective solubilization of correctly folded proteins and better preserve native lipid interactions than other membrane mimetics. Our spectra reveal the correlation between the post-translation modifications (PTMs), lipid-interactions, and protein-folding states of bR, providing insights into the process of maturation of the photoreceptor proteins.

## Introduction

Integral membrane proteins account for around a quarter of the human proteome^1^ and represent approximately 60% of all known drug targets.^2^ They have important roles in cell–cell communication, transport of substrates, and energy generation. Complex synthesis and folding pathways are required for these proteins, because the nascent polypeptide chain that emerges from the ribosome must be threaded through the membrane before the final native conformation is achieved.

Nanoelectrospray ionization (nESI) enables protein mass spectra to be acquired while maintaining native conformations under non-denaturing conditions.^3,4^ Soluble and membrane-embedded proteins have been studied using native mass spectrometry (MS), allowing interrogation of post-translational modifications (PTMs), ligand interactions, oligomeric states, and associated lipids.^5,6^ For example, native MS results revealed the regulatory role of PTMs on the ligand affinity of glycoproteins.^7^ Recent studies have also shown that lipids can stabilize the dimer interface(s) of a range of membrane proteins.^8,9^

Biological membranes, found in every organism, are complex, dynamic, and heterogeneous—properties which pose a considerable challenge for the preparation of proteins for many biophysical methods. Although most purification protocols for membrane proteins require detergents, the use of these amphipathic molecules may perturb interactions with lipids or ligands that may be required for protein activity and stability.^10−15^

A range of discoidal lipid-based nanoparticles, in which proteins may be embedded, have been shown to be suitable for native MS. Nanodiscs, stabilized by membrane scaffold proteins (MSPs), have been analyzed by native MS using two different energy regimes. Under high-energy dissociation, membrane proteins can be released from the MSP Nanodiscs, but few protein oligomers and protein–lipid complexes are typically observed in the spectra.^16−18^ Under low-energy dissociation, membrane proteins released from MSP Nanodiscs have been found to carry a large number of associated lipids (e.g., 40–120), leading to overlapping and only partially resolved peaks from which the stoichiometries of the annular lipids may be deduced.^19−22^ More recently, chemical additives have been used to maintain the integrity of Nanodiscs in the gas phase. Nanodisc ions retaining two MSP molecules also display a partially resolved peak pattern.^23^

Detergent-free Lipodisqs (also known as SMALPs) are formed when hydrolyzed copolymers of styrene and maleic anhydride (SMA) are added to native cell membranes under controlled conditions.^24,25^ These discoidal nanoparticles are now finding widespread application in membrane biophysics, including for drug delivery, structural studies, purification applications, and spectroscopic investigations.^24,26,27^ Lipodisqs have been studied recently using a laser-induced liquid bead ion desorption (LILBID) coupled to a time-of-flight (ToF) mass spectrometer.^28^ LILBID yields low charge state ions at the high *m*/*z* range (e.g., 100,000 *m*/*z*), where the resolution is dramatically lower compared to the low *m*/*z* range. Furthermore, due to the limitation of the resolution of the ToF detector, protein–lipid complexes and Lipodisq ions observed in the spectra were largely unresolved. Nevertheless, it is possible to determine the oligomeric state of the proteins from the mass spectra.

Bacteriorhodopsin (bR) is a homotrimeric, 27 kDa membrane-embedded photoreceptor, expressed by the archaebacterium *Halobacterium salinarum*. It harnesses the energy from sunlight to generate a transmembrane H^+^ ion gradient for ATP synthesis.^29,30^ The protein is synthesized as bacterio-opsin (28256 Da, UniProtKB: P02945) and its maturation requires several PTMs: (i) the signal peptide (SP, corresponding here to the first 13 N-terminal amino acids) is removed and Gln14 is modified to a pyroglutamate residue (or pyrrolidine carboxylic acid, abbreviated PCA).^31^ The PCA residue may mediate the interaction between receptors or protect against N-terminus degradation in the native membrane;^32,33^ (ii) Asp262 at the C-terminus is also removed, however, the purpose of this cleavage is unclear. (iii) Retinal is covalently conjugated to Lys216 via a Schiff base linkage to create the retinylidene chromophore, enabling bR to absorb light.^34^ The precise sequence and coordination of these modifications have not yet been determined.

Here, we present a native MS strategy to characterize the PTMs and lipid interactions of bR and its homolog archaerhodopsin-3 (AR3) using proteins prepared using Lipodisq, MSP Nanodisc, and detergent-based protein extraction methods. We observe well-resolved MS spectra from Lipodisq and MSP Nanodiscs, consistent with the ejection of the protein from the nanoparticle in the gas phase. The energy required for the ejection is lower for the Lipodisq than for MSP Nanodiscs. We also show that SMA selectively extracts the fully mature bR protein, whereas detergents also solubilize a range of immature isoforms. By comparing all three extraction methods, we are able to correlate the extent to which the protein is folded with truncations of the SP, helping to further our understanding of post-translational membrane protein processing.

## Materials and Methods

### Native Mass Spectrometry

bR and AR3 were purified from their native membranes and prepared for mass spectrometry (see Supporting Information). The photoreceptors, in either detergent, Lipodisqs, or Nanodiscs, were buffer-exchanged into 0.2 M ammonium acetate, pH 7 (containing 2 × CMC OG for detergent samples), before native MS analysis. Mass spectra were acquired with a Q-Exactive Plus Hybrid Quadrupole-Orbitrap Mass Spectrometer^35^ following an established protocol.^36^ In-source trapping (IST) voltages above 20, 60, and 80 V were applied to liberate photoreceptors from the detergent micelles, Lipodisqs, and MSP Nanodiscs, respectively. For tandem MS experiments, the quadrupole was operated with a 5 *m*/*z* isolation window width to isolate protein–lipid complexes for further collisional activation in the HCD cell (0–100 V). Spectra were acquired from at least two biological replicates. Observed masses and uncertainties between charge states were determined using an Excel script kindly provided by the Benesch Laboratory, University of Oxford.^37^

### Peak Assignment

Lipid adducts and PTMs were confirmed using tandem MS and denaturing MS. The masses of the lipid adducts were determined by native MS and were identified using the LipidMAPS and PDB databases. PTMs were assigned based on exact masses and previously reported PTMs of bR.^34,38−43^

## Results and Discussion

### Native Mass Spectra of Protein Lipodisqs Are Well Resolved

**Figure 1:**
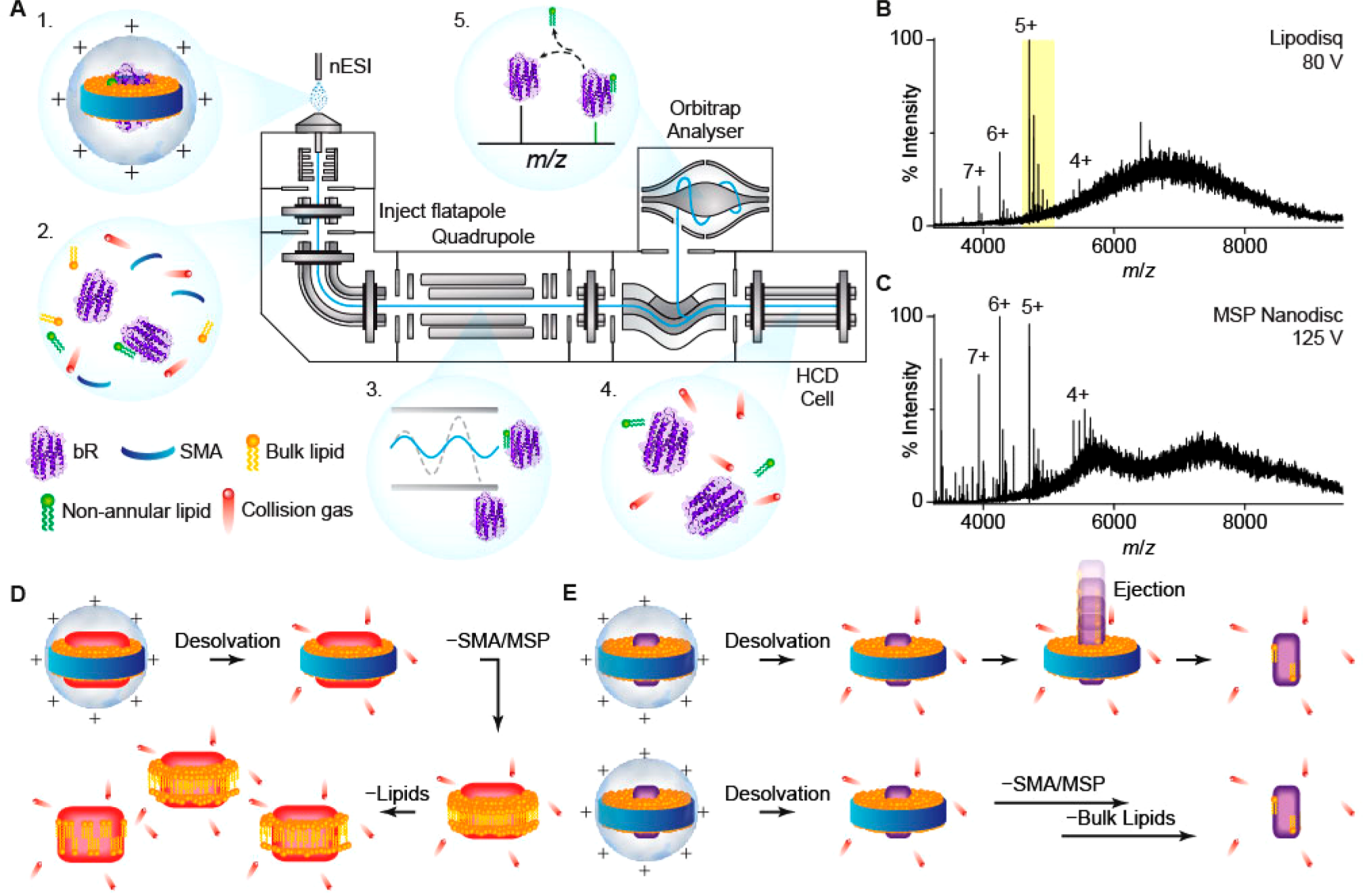
(A) Schematic of a tandem MS experiment, performed in a high-resolution Q-Exactive Plus mass spectrometer. (1) Lipodisqs are ionized by nESI and (2) activated in the inject flatapole to release the membrane protein. (3) A single protein–lipid complex can be isolated by the quadrupole and (4) further activated in the HCD cell to dissociate complexes, (5) revealing the composition of the complex. (B) and (C) are representative spectra of bR in Lipodisq and MSP Nanodisc preparations. The 5+ charge state of the liberated bR highlighted in (B) is expanded in Figure 2B and 3A. (D) Lipoprotein nanoparticles with large embedded proteins have been shown to gradually dissociate from MSPs and lipids before releasing the reconstituted protein, leading to many intermediates of protein–lipid complexes. (E) Small proteins are released from the nanoparticles with few lipids, possibily due to a direct ejection from the intact Lipodisqs/Nanodiscs (top) or a rapid loss of bulk lipids following SMA/MSP dissociation (bottom).

bR reconstituted in Lipodisqs, prepared without the use of detergent, or in proteoliposomes were characterized by dynamic light scattering, by circular dichroism, and by their absorbance spectra (Figure S1). We prepared bR or AR3 in MSP Nanodiscs, Lipodisqs, and detergent micelles. These samples were ionized by nESI and analyzed using a high-resolution Orbitrap mass spectrometer (Figure 1 A).^44^ Well-resolved *apo*-protein and adducts appeared at a collision voltage of 80 V for both bR and AR3 (Figures 1 B, 3A, and 3B). These spectra are surprisingly different from previously reported spectra of MSP Nanodiscs and Lipodisqs^19,28^ in that the peaks are remarkably well-resolved with few associated lipids even at relatively low energies.

Spectra of bR reconstituted in MSP Nanodiscs (Figure 1 C) are qualitatively similar to the well-resolved spectra of bR-Lipodisq (Figure 1 B) and show identifiable protein and adduct peaks. Intriguingly, both spectra lack the overlapping peak pattern observed in the MSP Nanodisc spectra of other proteins.^16^ The gas-phase disruption of MSP Nanodiscs has been suggested to be initiated with the dissociation of the MSPs, followed by a stepwise release of lipids from the *apo*-protein (Figure 1 D).^19^ As lipids dissociate gradually, many dissociation intermediates are observed, which originate from the number and diversity of the associated lipids, and give rise to the overlapping peaks in the spectra. The lack of overlapping peaks in our spectra implies a direct ejection of bR from the intact Lipodisqs/Nanodiscs (Figure 1 E, top). We suggest this is possible because bR is significantly smaller than the proteins studied previously (i.e., >100 kDa) and interacts with fewer lipids. However, we do not exclude the possibility that bulk lipids dissociate immediately after desolvation and dissociation of SMA/MSP (Figure 1 E, bottom).

The collision voltage required to eject bR from Lipodisq is lower than that for MSP Nanodiscs. We suggest that additional energy is required to dissociate the MSP, which forms α-helical coils around the rim of the Nanodisc and which is stabilized by intramolecular H-bonds. In constrast, no significant internal H-bonding is thought to take place in SMA.^24^ Although Nanodiscs with pure phosphatidylglycerol or phosphatidylserine can be ionized in positive polarity mode,^21^ these anionic lipids must be protonated during ionization to achieve a net positive charge on the Nanodisc ion. SMA contains numerous titratable carboxylic acid groups, and if the pH of a suspension of Lipodisq nanoparticles is lowered, these negatively charged groups become protonated and the polymer dissociates from the lipids and precipitates.^24^ We propose that this protonation also takes place upon ionization of Lipodisqs to produce a nanoparticle with a net positive charge, thereby facilitating SMA dissociation and reducing the collision voltage required to liberate the protein relative to other nanoparticles. This feature makes Lipodisqs attractive for native MS and applicable for tandem MS strategies, since lower energy is required to release proteins prior to the quadrupole.

### bR Signal Peptide Cleavage Follows Schiff Base Formation

In order to study the PTMs of bR, the protein was also prepared for native MS by *n*-octyl-β-d-glucoside (OG) detergent extraction. bR retains its secondary structure when extracted with either OG^45,46^ or SMA as shown by circular dichroism spectroscopy (Figure S1C). By comparing the mass spectra of the same protein produced using different methods, we aimed to determine not only the exact mass of mature bR and its precursors but also to characterize interactions with endogenous lipids.

**Figure 2:**
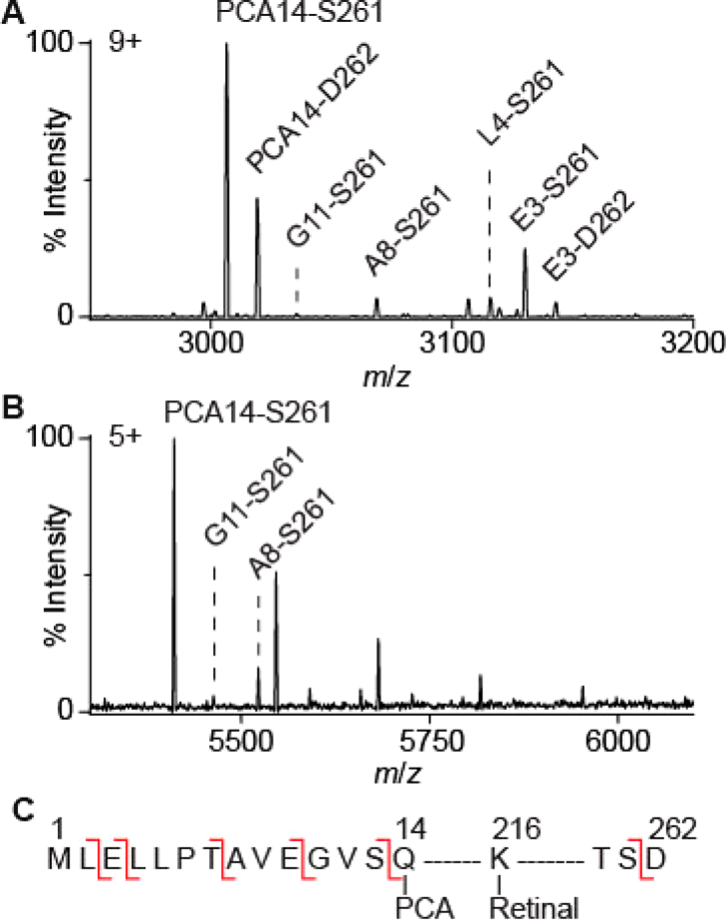
Different proteoforms are observed for monomeric bR solubilized with OG (A) and extracted with SMA (B). The observed PTMs are indicated on the amino acid sequence of bacterio-opsin (C). These PTMs include SP truncations (before E3, L4, A8, G11, Q14), conversion of N-terminus Q14 to PCA, removal of D262, and retinal Schiff base formation (present in all observed proteoforms). Note that SP truncation (red lines) takes place *in vivo* and is not the result of gas-phase fragmentation which does not occur under these MS conditions.

The most prominent species (molecular weight of 27049 ± 1 Da, Table S1) detected in both OG- and SMA-solubilized bR corresponds to fully mature bR (PCA-A_15_···S_261_)^40,47^ (Figure 2 A, B). A number of additional adducts (PCA-A_15_···D_262_, L_4_···S_261_, E_3_···S_261_, and E_3_···D_262_) were observed for OG-solubilized preparations that were absent in SMA-solubilized preparations. These adducts could not be dissociated by tandem MS (Figure 1 A), confirming that they are covalent modifications. Denaturing mass spectra acquired in organic solvent retained these modifications but revealed the absence of noncovalently bound endogenous ether-linked lipid (2,3-di-O-phytanyl-*sn*-glycero-1-phospho-(3′-*sn*-glycerol-1′-methyl) phosphate, PDB ID: 2DP, Figure S2).

bR is encoded by the *bop* gene, which corresponds to the unmodified precursor protein bacterio-opsin^43^ with a predicted mass of 28256 Da. Bacterio-opsin was not observed in any of our preparations, and this unprocessed form has not previously been shown to be present in native purple membranes.^38,48,49^

The first part of bacterio-opsin to be produced during translation is the signal or leader peptide (SP), which directs the ribosome to the membrane, controls the kinetics of protein folding, and reduces misfolding.^50,51^ N-terminus sequencing data suggest that the SP cleavage of bR is a complex, multistage process with at least four distinct cleavage events^49^ in either sequential or parallel pathways and that some of the immature protein is incorporated into the native purple membrane.^49^ From the masses observed, we deduce that all of the proteoforms identified were already covalently conjugated to retinal, implying that this step takes place early in the maturation process. The reasons behind the relatively large number of cleavage sites in the SP are unclear; however they may relate to stages in the oligomerization of the protein in the highly ordered photoactive membrane of the native organism.

We correlated the covalent adducts observed in our bR spectra (Figure 2, Table 1) with immature forms in which the SP has been partially cleaved. Cleavage sites at the N-terminal side of Leu4, Ala8, Gly11, and Gln14 were identified, consistent with previous work using Edman Degradation and denaturing MS^38^ (Figure 2 C, Table 1). In OG-solubilized bR, an extra cleavage site at the N-terminal side of Glu3 was observed, which has not been previously reported.^38,48,49^ These isoforms are not present in the Lipodisq preparations. Some of the PTMs of bR that we identified include the removal of Asp262 at the C-terminus.

**Table 1:** Different Isoforms of bR Observed by Denaturing^38^ and Native MS (This Study)

First residue	Protein sequence	Edman Degradation denaturing MS^38^	Detergent-containing native MS^d^	Lipodisq/MSP Nanodisc native MS
Met1	M_1_L-E-L-LP-T-AVE-GVS-QA_15_···D_262_	X	X	X
Glu3	E-L-LP-T-AVE-GVS-QA_15_···D_262_	X	present	X
Leu5	LP-T-AVE-GVS-QA_15_···D_262_	present^b^	X	X
Thr7	T-AVE-GVS-QA_15_···D_262_	present^b^	X	X
PCA14	PCA-A_15_···D_262_	X	present	X
Glu3	E-L-LP-T-AVE-GVS-QA_15_···S_261_	X	present	X
Leu4	L-LP-T-AVE-GVS-QA_15_···S_261_	present^b^^,^^c^	present	X
Ala8	AVE-GVS-QA_15_···S_261_	present^b^	present	present
Gly11	GVS-QA_15_···S_261_	present^b^^,^^c^	present	present^e^
PCA14	PCA-A_15_···S_261_	present^a^^,^^f^	present	present

a Under denaturing conditions, only the PCA-A_15_···S261 isoform has been observed previously with the retinal Schiff base; all other isoforms were observed without retinal. The unmodified bacterio-opsin precursor protein was not observed either in previous studies or by our MS method.

b Observed without covalently bound retinal.

c Tentative assignment by Barnidge et al.^38^

d The same species are observed for denaturing or native MS (Figure 2A and Figure S2).

e The presence of the proteoform cannot be confirmed in MSP Nanodiscs due to low abundance and baseline noise.

f Following cleavage at the C-terminal side of Ser13, Gln14 undergoes a cyclization reaction to become pyrrolidine carboxylic acid (PCA) which lacks a primary amine group.

### Extraction of bR by SMA Preserves Native Lipid–Protein Interactions That Are Disrupted by Detergents

**Figure 3:**
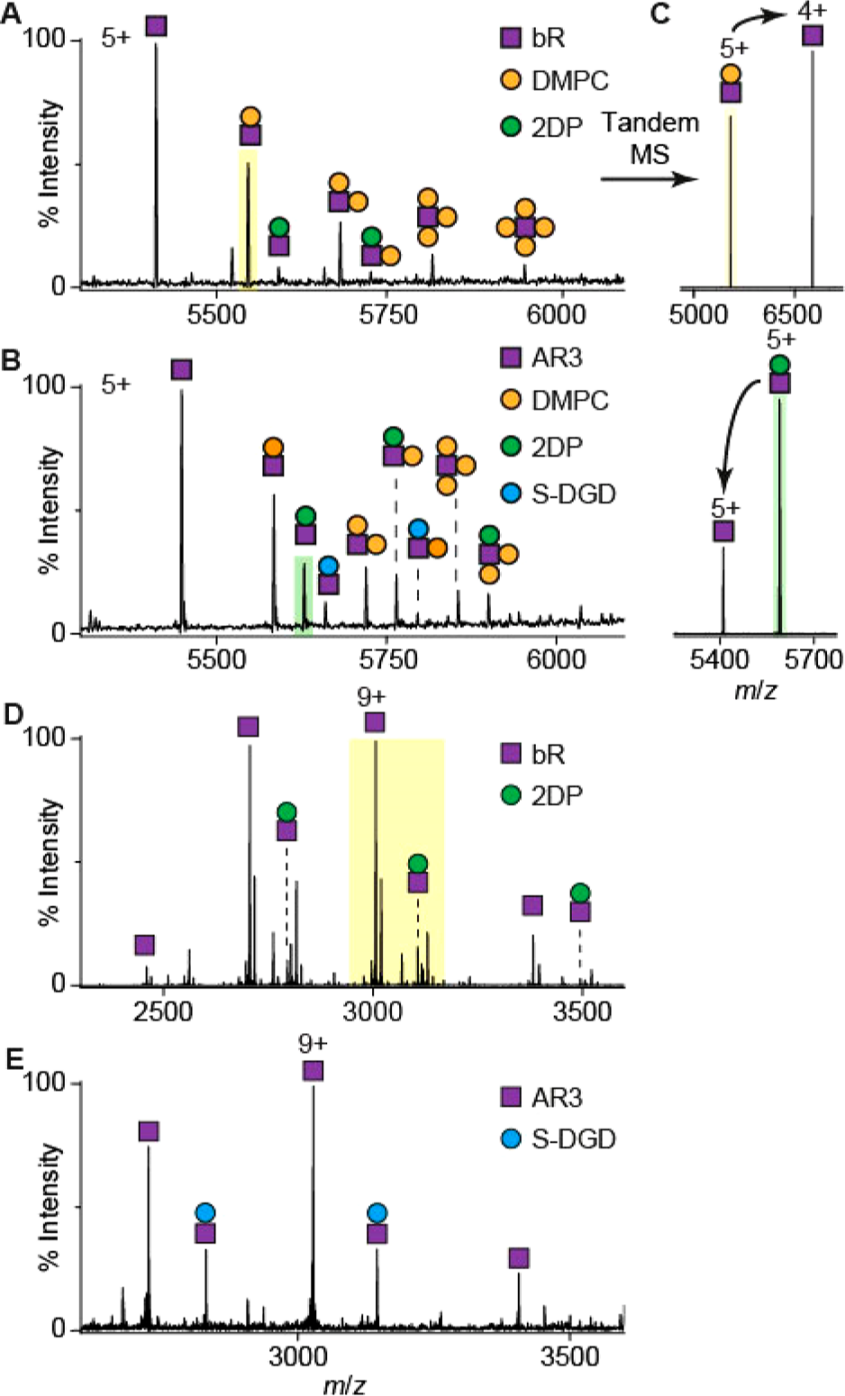
(A) bR and (B) AR3 are dissociated with a number of associated lipids from Lipodisqs in the gas phase. Tandem MS spectra of shaded peaks in (A) and (B) are shown in (C). (C) Tandem MS is used to confirm any noncovalent interactions and complex composition. The charge state of the dissociated lipid, determined by its headgroup, affects the charge state of the stripped *apo*-protein. An interaction with 2DP is also observed for OG-solubilized bR (D) but is lost in AR3 (E). The yellow shaded region in (D) corresponds to the spectra shown in Figure 2A.

bR can be dissociated from Lipodisq nanoparticles by nESI in association with noncovalently bound lipids (Figure 3 A). In addition to the covalent adducts discussed above, two noncovalent adducts are identified in the spectra. The adduct of mass 678 ± 1 Da corresponds to exogenous DMPC lipid (orange dot), which is added with SMA polymer during the extraction procedure.^52^ The second adduct, with a mass of 901 ± 1 Da (green dot), corresponds to an endogenous ether-linked lipid, 2DP, found extensively in the native purple membrane. Several crystal structures of bR also include this lipid,^53^ which has previously been shown to be important for the stability and activity of many microbial rhodopsins.^54,55^

Spectra of Lipodisqs containing AR3, a homolog (59% amino acid identity) of bR from the related archaea *Halorubrum sodomense*,^56,57^ revealed the presence of an additional lipid adduct, 1-O-(6′′-sulfo-α-d-mannosyl-1′′-2′-α-d-glucosyl)-*sn*-2,3-di-O-phytanylglycerol (S-DGD, 1057 ± 1 Da, Figure 3 B, blue dot).^57−59^ S-DGD lipids enable the lipid bilayer to withstand variations in osmolarity,^60,61^ since the C20 (and C25) lipid chains make the bilayer thicker and more resilient against changes in pH and salt concentration.^62^ Some reports speculate that these lipids could act as proton donors for ion transport by microbial rhodopsins.^63^

Tandem MS was used to further validate the lipid assignments by collision-induced dissociation (CID) of the protein–lipid complexes leading to stripped *apo*-proteins and lipid adducts (Figure 1 A).^36^ This approach differentiates between noncovalent and covalent interactions, since the latter require a much higher activation energy to fragment. Tandem MS data confirm that the protein–lipid complexes are noncovalent, and in positive polarity mode, the lipid adducts dissociate as cationic or neutral species leading to *apo*-protein ions with reduced or identical charge states (Figure 3 C).^21^

We next compared our spectra obtained from Lipodisqs with those obtained for detergent-purified bR and AR3 (Figure 3 D, E). We observed 2DP association for bR and S-DGD association for AR3 as observed in the Lipodisq spectra. However, 2DP interactions were lost in OG-solubilized AR3, most likely since the lipid was displaced by the detergent. The ability to observe native lipid adducts is highly dependent on the solubilization/extraction conditions.^64^ Lipodisqs are prepared without the use of detergents, and native lipid–protein interactions are more likely to remain intact in the nanoparticle environment.

The major charge state of bR and AR3 observed in the Lipodisq mass spectra (5+) is lower than that from OG micelles (9+) (Figure 3 D, E). Although it is unlikely that non-ionic OG produces a significant supercharging effect, the charge states observed for proteins in saccharide detergents generally agree with the theoretical Rayleigh limit but are higher than other classes of detergents, e.g., polyethylene glycol detergents and amine oxide detergents.^65^ Furthermore, DMPC phosphate groups may accept protons and become positively charged. In our tandem MS (Figure 3 C), a charge-reducing effect was observed for DMPC adducts but not for the highly anionic ether lipid aducts.^66^ We propose that dissociation of DMPC molecules, with a net positive charge, is also responsible for the lower charge state of bR observed in the Lipodisq spectra compared with the OG micelles.

### Lipodisq Nanoparticles Selectively Incorporate Fully Folded Membrane Proteins

In order to extract bR from its native membrane using SMA, DMPC is added to facilitate Lipodisq nanoparticle formation.^52^ This exogenous lipid is known to favor the incorporation of correctly folded bR over partially folded forms.^52,67−71^ The absence of immature proteoforms in bR-Lipodisq spectra may relate to the immature protein adopting an incompletely folded structure.

**Figure 4:**
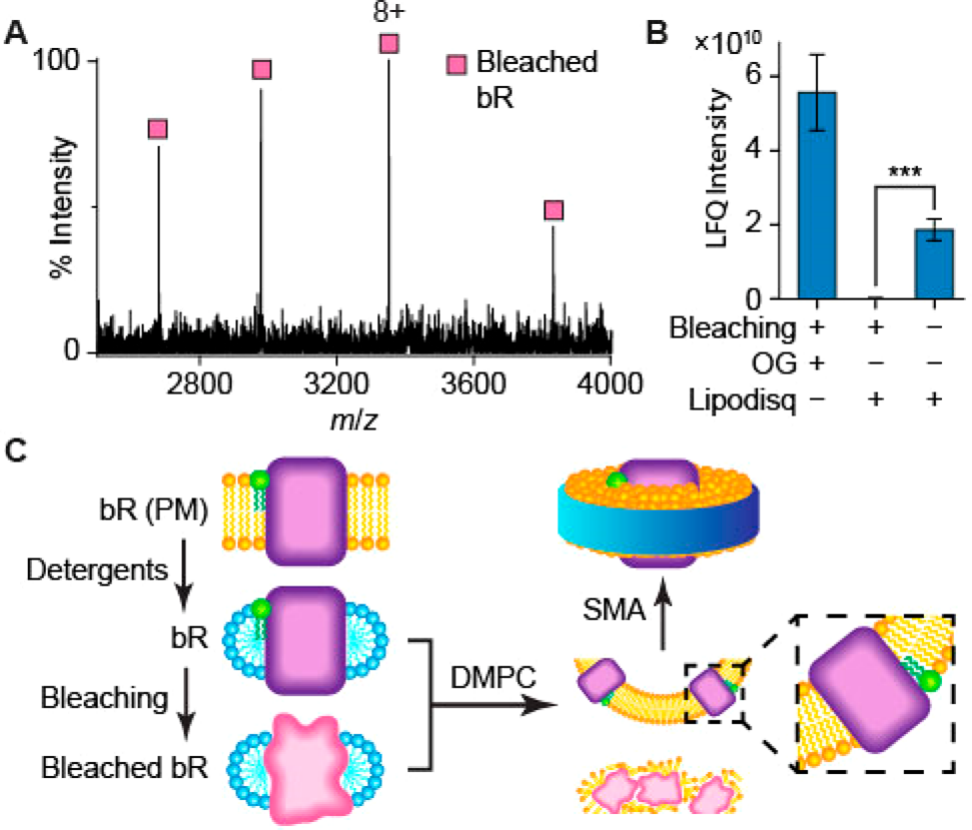
(A) The charge state distribution of bleached bR in OG detergent, observed in native MS spectra, indicates that the protein is folded in solution, but no associated 2DP lipid is observed. (B) Proteomics was used to quantify the amount of chemically bleached bR, before and after Lipodisq reconstitution, using label-free quantification (LFQ) of intensities, following in-gel digestion (*n* = 3, ***P < 0.001). Starting with equal amounts of protein, chemically bleached bR is not observed after Lipodisq reconstitution, whereas retinal-bound bR is detected readily. (C) A schematic showing that both properly folded and misfolded proteins can be retained in detergents; however, only the fully folded species was incorporated into the lipid bilayer of Lipodisqs.

In the absence of retinal, immature bR is present as a partially folded intermediate.^67,68,70^ We propose that bR without retinal that had not reached its final, fully folded conformation, would not be amenable to reconstitution in Lipodisq nanoparticles. To test this hypothesis, OG-solubilized bR was chemically bleached with hydroxylamine^30,72^ prior to extraction with SMA. The bleached bR (26782 ± 1 Da), lacking retinal, was detectable in the OG-solubilized preparations by both native MS and proteomics analysis (Figure 4 A, B) but not in the Lipodisq preparations (Figure 4 B).

Since the charge state distribution of a protein in nESI is correlated with its folded state and surface area, unfolded proteins have higher charge states than folded proteins, which have smaller surface area.^73^ Bleached bR exhibits a charge state distribution comparable to that of unbleached bR (Figure 4 A) and therefore retains a compact, if incompletely folded, structure. Furthermore, no interactions between 2DP and the bleached protein were observed, consistent with the disruption of the lipid association sites in the partially folded protein.

## Conclusion

In this study, we have demonstrated that native MS spectra of photoreceptor proteins, extracted from native membranes using SMA polymer, are well resolved and that both covalent and noncovalent adducts can be readily identified. bR and AR3 are released from Lipodisq nanoparticles at lower collison voltages compared to MSP Nanodiscs. We suggest that the energy required may be dependent on the size of the embedded protein. The proteins released from nanoparticles have a lower charge state than those released from detergent preparations, probably resulting from the charge-reducing effect of the dissociation of DMPC.

We have used both detergent-facilitated and detergent-free approaches, in combination with native MS, to characterize the PTMs of bR. We are able to identify five distinct cleavages of the signal peptide at the N-terminus and show, for the first time, that the covalent conjugation of retinal to Lys216 precedes these truncations.

The addition of DMPC during nanoparticle formation does not perturb the interactions between bR and the ether lipid 2DP. The binding of 2DP to bR has previously been observed by other biophysical methods,^74^ including X-ray crystallography.^75^ A second native lipid adduct, S-DGD, in addition to 2DP, is identifiable in the Lipodisq spectrum of AR3. However, only S-DGD is observed in the OG spectrum of AR3. Our data therefore confirm that protein extraction from native membranes by SMA polymer does not disrupt the noncovalent binding of native lipids and suggests that Lipodisq-MS will be a useful tool in the study of specific protein–lipid interactions.^19,28^ Lipodisq MS may also assist in the identification of different lipids and/or ligands present in crystal structures.^76^

Finally, we have shown that bR which is not in its final native conformation (e.g., chemically bleached bR from which retinal has been removed, and possibly the immature bRs) is present in detergent-solubilized preparations but absent in SMA-solubilized preparations, suggesting that this methodology is selective for correctly folded membrane proteins. We foresee that combining Lipodisq and native mass spectrometry will play a key role in the study of membrane protein ligand binding, maturation, and folding.

## Supporting Information Available

Method of protein expression and purification; bleaching of bR; SMA hydrolysis; Lipodisq/Nanodisc reconstitution; denaturing MS; proteomics; UV–vis spectroscopy; DLS; CD; and Table S1 and Figures S1 and S2 (PDF)

## Abbreviations

2DP: 2,3-di-O-phytanyl-*sn*-glycero-1-phospho-(3′-*sn*-glycerol-1′-methyl phosphate)
AR3: archaerhodopsin-3
bR: bacteriorhodopsin
CD: circular dichroism
CID: collision-induced dissociation
DMPC: 1,2-dimyristoyl-*sn*-glycero-3-phosphocholine
IST: In-source trapping
LILBID: laser-induced liquid bead ion desorption
MS: mass spectrometry
MSP: membrane scaffold protein
nESI: nano–electrospray ionization
OG: *n*-octyl-β-d-glucoside detergent
PCA: pyrrolidine carboxylic acid
PTM: post-translational modification
S-DGD: 1-O-(6′′-sulfo-α-d-mannosyl-1′′-2′-α-d-glucosyl)-*sn*-2,3-di-O-phytanylglycerol
SMA: hydrolyzed copolymer of styrene and maleic anhydride
SP: signal peptide
ToF: time of flight.
