# Supplementary material for: Detergent-free Lipodisq Nanoparticles Facilitate High-Resolution Mass Spectrometry of Folded Integral Membrane Proteins
Source: Nano Lett. 2021 Mar 31;21(7):2824–31. doi: 10.1021/acs.nanolett.0c04911 (PMC8050825; doi:10.1021/acs.nanolett.0c04911)
Supplement: Supplementary file 1 — nl0c04911_si_001.pdf [file nl0c04911_si_001.pdf]

Supporting Information

Detergent-free Lipodisq nanoparticles facilitate high-resolution mass spectrometry of folded integral membrane proteins

Kin Kuan Hoi‡[1], Juan Francisco Bada Juarez‡[2], Peter J. Judge‡[2], Hsin-Yung Yen[1,3], Di Wu[1], Javier Vinals[2], Garrick F. Taylor[2]; Anthony Watts[2*] and Carol V. Robinson[1*]

[1]Department of Chemistry, Physical and Theoretical Chemistry Laboratory, University of Oxford, South Parks Road, Oxford, OX1 3QZ, United Kingdom.

[2]Department of Biochemistry, Biomembrane Structure Unit, University of Oxford, Oxford, OX1 3QU, United Kingdom.

[3]OMass Therapeutics, The Schrödinger Building, Oxford Science Park, Oxford, OX4 4GE, United Kingdom

MATERIALS AND METHODS

Bacteriorhodopsin expression and purification *Halobacterium salinarum* starter cultures were inoculated in a modified Peptone medium (LP72, Oxoid) and cultured up to an OD_{600} of 1.8-2.0 (5-7 days). Conical flasks (2 L) each containing 500 mL of media were inoculated with 25 mL of starter culture. Growth was allowed to proceed at 37 °C, 150 rpm under light illumination (30 W LED white light) up to an OD_{600} of 2-2.1 (12-15 days). Cells were harvested by centrifugation (8000 ×*g*, 30 min, 4°C). Pellets were resuspended in 20 mL of 4 M NaCl. DNase (2 mg) was added and stirred for 10 min at 4°C. The suspension was homogenized using a 10 mL homogenizer and dialyzed overnight in 0.1 M NaCl. The membranes were centrifuged (50,000 ×*g*, 1h, 4°C) and the pellet was resuspended in ddH_2O and applied to a sucrose gradient of three different layers (20%, 40% and 60% w/v sucrose). Sucrose gradient was centrifuged (110,000 ×*g*, 16 h, 15 °C). The purple layer was collected and dialysed against water in order to remove the excess of sucrose. The purple membrane was then centrifuged (40,000 ×*g*, 45 min, 4 °C) and the pellet resuspended in ddH_2O.

Expression and purification of AR3 *Halorubrum sodomense* strain RD-26 (ATCC-33755) cells were purchased from LGC Standards Ltd (Teddington, UK) and were grown according to the manufacturer's protocol with some minor modifications. Initially cells were grown in 20-25 mL of liquid culture media with high salt concentration (125 g NaCl, 160 g $MgCl_2$, 0.13 g $CaCl_2$, 5 g K_2SO_4, 1 g bacteriological peptone (LP72, Oxoid), 1 g yeast extract, 2 g soluble starch) pH 7.4 at 45 °C and 170 rpm shaking for 5-7 days (until the OD_{600} reached 1.2). Flasks containing 1 L of fresh culture medium were inoculated from the 25 mL cultures and incubated at 45 °C and 170 rpm shaking for at least 12 days. Cells were harvested by centrifugation after the OD_{600} had reached 1.7-1.9 (8000 ×*g*; 30 min; 4 °C) and the pellets were resuspended in 4 M NaCl, mixed together, stirred for 2 h or manually homogenized, and then 5 mg of DNAse (Sigma) was added for 30 min at 4 °C. The preparation was dialyzed overnight in 0.1 M NaCl, and centrifuged (70,000 ×*g*; 45 min; 4 °C). Sucrose density gradient ultracentrifugation was used to isolate the AR3-rich claret membrane, using a step gradient of 35 %, 40 %, 45 % and 50 % w/v, centrifuged (110,000 ×*g*, 15 h, 15 °C). The bottom band with a pink/purple colour was collected. Dialysis was performed against MilliQ water overnight in order to remove the sucrose. The sample was then further centrifuged (70,000 ×*g*, 45 min, 4 °C), and the pellet was resuspended in ddH_2O to a final concentration of 20 mg/ml and stored at 4 °C prior to spectroscopic experiments.

Delipidation of the microbial rhodopsins Proteins were delipidated following literature protocols[1,2]. The protein-containing membranes were pelleted by centrifugation (70,000 ×*g*, 30 min, 4 °C), and resuspended in 6 mL of 25 mM NaH_2PO_4, pH 6.9. Detergent (n-octyl-β-D-glucoside (OG), Glycon) was added to the sample (2 mL of 10% w/v OG in ddH_2O pH 7) and the preparation was sonicated for 1 min in a bath sonicator at room temperature. The sample was incubated at 22 °C overnight without stirring. The solution was adjusted to pH 5.5, and centrifuged (100,000 ×*g*, 45 min, 15 °C) to remove aggregates and any non-solubilised material. The supernatant was applied to a preparative gel column (Hi-Load 16/600, Superdex 200 pg) pre-equilibrated in 25 mM NaH_2PO_4, 1.2% w/v OG at pH 5.5. Fractions were collected from chromatogram peaks and further analysed. Colored fractions were further concentrated using a VivaSpin concentrator to 9 mg/mL and stored at 4 °C.

Bleaching of bacteriorhodopsin (removal of retinal from bR) Detergent-solubilised bR was bleached by adding 0.2 M hydroxylamine (pH 7) under a halogen lamp. The solution was stirred on ice for 3 h until it became colourless. The protein was dialysed in buffer (25 mM NaH_2PO_4, 1.2% w/v OG at pH 5.5) to remove hydroxylamine.

SMA hydrolysis. A polymer of styrene and maleic anhydride (in a 3:1 molar ratio) was kindly provided by Malvern Cosmeceutics. The polymer was hydrolysed to produce SMA by adding 1 M NaOH solution (5% w/v final) (Fisher), heated at 80-90 °C for 1-2 h. The solution turned from cloudy to clear yellow. 5 M HCl was then added to precipitate the SMA,

which was pelleted by centrifugation (2000 ×*g*, 5 min, RT). The pellet was resolubilised in water and was subjected to multiple washing and centrifugation steps (2000 ×*g*, 5 min). Once washed, ddH_2O was added to the solution and dialysis was performed overnight in order to remove the excess of salt and to adjust the pH. The resulting pale yellow, transparent SMA solution was lyophilized. The solid white powder was redissolved in ddH_2O or buffer to a final concentration of 125 mg/ml and the solution adjusted to pH 8.

Solubilizaton of bR in OG and reconstitution of bR into MSP Nanodiscs. Proteins were delipidated following published protocols[1,2]. Briefly, the protein-containing membranes were pelleted by centrifugation (70,000 ×*g*, 30 min, 4 °C), and resuspended in 6 mL of 25 mM NaH_2PO_4, pH 6.9. Detergent (n-octyl-β-D-glucoside (OG), Glycon) was added to the sample (2 mL of 10% w/v OG in ddH_2O pH 7) and the preparation was sonicated for 1 min in a bath sonicator at room temperature. The sample was incubated at 22 °C overnight without stirring. The solution was adjusted to pH 5.5, and centrifuged (100,000 ×*g*, 45 min, 15 °C) to remove aggregates and any non-solubilized material. The supernatant was applied to a preparative gel column (Hi-Load 16/600, Superdex 200 pg) pre-equilibrated in 25 mM NaH_2PO_4, 1.2% w/v OG at pH 5.5. Fractions were collected from chromatogram peaks and further analyzed. Colored fractions were further concentrated using a VivaSpin concentrator to 9 mg/mL and stored at 4 °C.

OG-solublized bR was reconstituted following an established protocol[3]. Briefly, purifed bR, MSP, and DMPC solubilized in cholate, were mixed at 1:2:80 ratio and incubated at room temperature for 2 h. After addition of amberlite XAD-2 hydrophobic beads, the mixture was incubated overnight. MSP Nanodiscs containg bR were purified on a Superose 6 increase 10/300 column, equilibrated in 0.2 M ammonium acetate, pH 7.

Incorporation into Lipodisq nanoparticles. bR from purple membrane and AR3 from claret membranes, were incorporated separately into Lipodisqs as described previously[4] with some changes outlined below. Briefly, 4 mg of protein was pelleted (45,000 ×*g*, 30 min, 4°C) and resuspended in 1 mL of 50 mM sodium phosphate and 200 mM NaCl buffer containing 20 mg of 1,2-dimyristoyl-*sn*-glycero-3-phosphocholine (DMPC) small unilamellar vesicles (SUVs) produced by the freeze/thaw and extrusion technique in a final protein/lipid molar ratio of 1:172. The solution was sonicated (30 min) in a bath sonicator with gentle heating (30-35 °C). Styrene-maleic acid copolymer (25 mg, added as a solution in ddH_2O) was added to the vesicle membrane sample, and the solution incubated (42 °C, 1 h). Samples were centrifuged (100,000 ×*g*, 30 min, 15 °C) in order to remove non-solubilized membrane and the supernatant collected.

Reconstitution of detergent-bR into Lipodisqs using proteoliposomes. The protocol was adapted from[5,6]. Briefly, DMPC was purchased from Avanti Polar Lipids. The appropriate amount of lipid was dissolved in chloroform:methanol (50:50) to 10-25 mg/mL in a round-bottom flask. The lipid solution was dried down to a lipid film either under nitrogen. The film was dried further overnight in a desiccator under vacuum and stored at -20 °C until needed or used immediately.

The lipid film was suspended in liposome buffer (50 mM sodium phosphate and 200 mM NaCl, pH 8) to give a final concentration of 5 mg/mL, and sonicated (3×1 min) using a bath sonicator, followed by 10 freeze-thaw cycles using liquid nitrogen and a 37 °C water bath. The lipid solution was then extruded through a 100 nm polycarbonate filter using a mini-extruder, for at least 11 passes to obtain a homogeneous distribution of liposomes of 100 nm in diameter. OG was added to the lipid suspension at a final concentration of 25.6 mM and the lipids were gently stirred for 0.5-1h (RT). The detergent-liposome mix was then added to bR (or bleached bR) at the desired lipid-to-protein ratio (172:1), and incubated for 30 minutes at room temperature.

Detergent removal was acheived in two steps. First, wet Bio-Beads (BioRad) (80 mg/mL) were added directly to the bR-lipid-detergent suspension. The mixture was incubated with light stirring at room temperature for 3 hr. A second portion of slightly wet beads was then added and mixed overnight with a small shaker to remove residual detergent. Bio-Beads were then removed, and proteoliposomes were harvested by centrifugation (100,000*g*, 1 h, 15 °C). The supernatant was removed and the pelleted proteoliposomes resuspended in buffer. To form Lipodisqs, SMA in a ratio of 1:1.5 (lipid-to-polymer w/w ratio) was added, and the solution incubated (37°C, 30 mins). Samples were centrifuged (100,000 ×*g*, 30 min, 15°C) in order to remove non-solubilized membrane and the supernatant collected. SMA was removed either by size-exclusion or by Vivaspin concentrator (Merck, MWCO 100 kDa).

Denaturing MS. Denaturing MS was performed following a well-established protocol with minor modifications[7]. Briefly, intact membrane protein was analysed on an UltiMate 3000 UHPLC system (Thermo Fisher Scientific) coupled to an Exactive Plus EMR mass spectrometer (Thermo Fisher Scientific). Membrane protein samples were loaded and separated on a ProSwift RP-4H capillary monolithic column (250 mm, Thermo Fisher Scientific) with a binary buffer system. Buffer A was 0.1% formic acid in 100% H_2O and buffer B was 0.1% formic acid in 5% H_2O, 45% acetonitrile, and 50% isopropanol. A linear gradient from 0% to 100% Buffer B was applied to elute membrane proteins. The mass spectrometer was operated in full MS scan mode with a mass range of 300 to 2,000 *m/z*. Spectra were deconvoluted by UniDec to obtained the mass distribution[8].

Proteomics. The amount of protein in the solution before and after Lipodisq reconstitution was quantified by gel-based proteomics. Briefly, equal amount of samples (15 µL) were separated by SDS-PAGE. The target gel bands were sliced into small pieces and digested with trypsin as described previously[9] . The digested membrane proteins were analysed on a Dionex 3000 UHPLC coupled to a LTQ-Orbitrap XL mass spectrometer (Thermo Fisher Scientific) as described previously[10]. The

peptides were separated on a 75 µm × 15 cm Pepmap C18 column (Thermo Fisher Scientific). LTQ-Orbitrap XL was operated in data-dependent acquisition mode with one full MS scan followed by 5 MS/MS scans with collision-induced dissociation. Proteomics data was analysed using Maxquant (version 1.6.7.0)[11]. Protein quantification is based on LFQ intensities. The means and errors were determined from three biological replicates.

Size-exclusion chromatography Lipodisq samples were applied to a Superdex Increase 200 10/300 GL column connected to an Akta Pure (GE Healthcare). The column was equilibrated with buffer for 2 column volumes and sample was applied at a flow rate of 0.5 mL/min. Protein was detected using a UV-detector at a wavelength of 280 nm and fractions were collected in 15 mL falcon tubes (Greiner). Purple fractions were collected and concentrated using a Vivaspin concentrator (Merck) and colourless fractions were discarded.

UV-Vis spectroscopy Spectra were recorded on a Jasco V-630 instrument at different wavelengths in the visible range, using a 1 cm quartz cuvette and were blanked against buffer.

Dynamic Light Scattering (DLS) Particle size was measured using a Malvern Zetasizer Nano S instrument in a disposable cuvette at 633 nm. The data was processed using Malvern Zetasizer software.

Circular dichroism (CD) CD spectra were acquired with a Jasco J-815 Spectropolarimeter using a 1 mm cuvette. A wavelength range (185-240 nm) was selected to monitor secondary structure of the proteins at ambient temperature. Concentration range used for measurements was adjusted between 0.01-0.1 mg/mL and the protein was diluted in low salt buffer in order to perform experiments. The data was analysed, and baseline corrected using the Spectra Manager software from Jasco. α, β and random coil content was calculated from the Dichroweb website[12] using the CDSSTR analysis programme[13] and the reference set SMP180 for membrane protein[14].

RESULTS

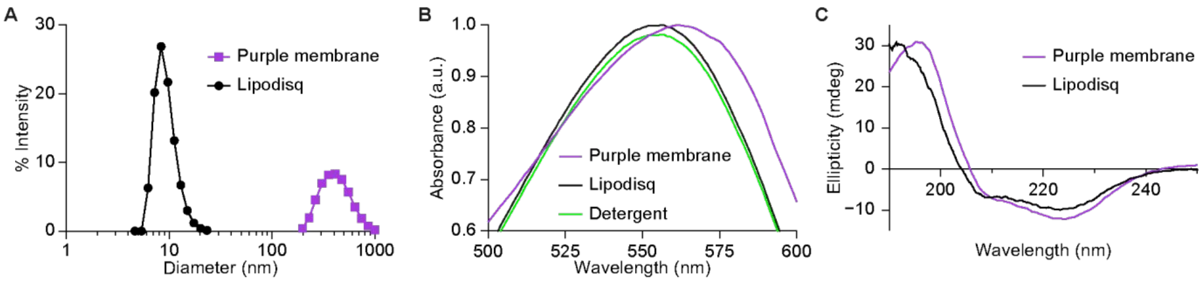

Figure S1. (A) Dynamic light scattering data for bR in proteoliposomes formed from the purple membrane by osmotic shock (purple) and in Lipodisq (black) with a measured size of 569 ± 245 nm and 9.5 ± 2.5 nm. (B) Absorption spectra of bR in purple membrane with A_{max} at 565 nm (purple), in OG detergent (green) and in Lipodisq with A_{max} at 555 nm (black), show a hypsochromic shift, characteristic of the change in oligomeric state from purple membrane (trimer) to Lipodisq (monomer)[4]. (C) UV CD spectra showing that the protein is folded both in the purple membrane (purple) and in Lipodisq nanoparticles (black).

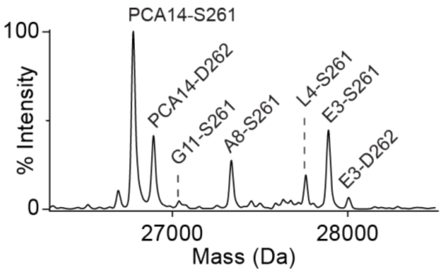

Figure S2. (A) Deconvoluted mass distributions of OG-solubilised bR, analysed under denaturing condition. Adducts present are covalent modifications.

Table S1. Theoretical and observed mass of photoreceptor .

Protein	Theoretical mass (Da)	Observed mass (Da)	Error (Da)
Mature bR (PCA-A_{15}……S_{261})	27,050	27,049	± 1
bacterio-opsin (PCA-A_{15}……S_{261})	26,784	26,782	± 1
AR3 (PCA-A_{8}……A_{257})	27,240	27,238	± 1

REFERENCES

(1) Nollert, P. Lipidic Cubic Phases as Matrices for Membrane Protein Crystallization. *Methods* **2004**, *34* (3), 348–353. https://doi.org/10.1016/j.ymeth.2004.03.030.

(2) Landau, E. M.; Rosenbusch, J. P. Lipidic Cubic Phases: A Novel Concept for the Crystallization of Membrane Proteins. *Proc. Natl. Acad. Sci.* **1996**, *93* (25), 14532–14535. https://doi.org/10.1073/pnas.93.25.14532.

(3) Ritchie, T. K.; Grinkova, Y. V.; Bayburt, T. H.; Denisov, I. G.; Zolnerciks, J. K.; Atkins, W. M.; Sligar, S. G. Chapter 11 Reconstitution of Membrane Proteins in Phospholipid Bilayer Nanodiscs. *Methods in Enzymology*. 2009. https://doi.org/10.1016/S0076-6879(09)64011-8.

(4) Orwick-Rydmark, M.; Lovett, J. E.; Graziadei, A.; Lindholm, L.; Hicks, M. R.; Watts, A. Detergent-Free Incorporation of a Seven-Transmembrane Receptor Protein into Nanosized Bilayer Lipodisq Particles for Functional and Biophysical Studies. *Nano Lett.* **2012**, *12* (9), 4687–4692. https://doi.org/10.1021/nl3020395.

(5) Dijkman, P. M.; Watts, A. Lipid Modulation of Early G Protein-Coupled Receptor Signalling Events. *Biochim. Biophys. Acta - Biomembr.* **2015**, *1848* (11), 2889–2897. https://doi.org/10.1016/j.bbamem.2015.08.004.

(6) Goddard, A. D.; Dijkman, P. M.; Adamson, R. J.; dos Reis, R. I.; Watts, A. Chapter Nineteen - Reconstitution of Membrane Proteins: A GPCR as an Example BT - Heterotrimeric G Proteins. In *Heterotrimeric G Proteins*; 2015.

(7) Toby, T. K.; Fornelli, L.; Srzentić, K.; DeHart, C. J.; Levitsky, J.; Friedewald, J.; Kelleher, N. L. A Comprehensive Pipeline for Translational Top-down Proteomics from a Single Blood Draw. *Nat. Protoc.* **2019**. https://doi.org/10.1038/s41596-018-0085-7.

(8) Marty, M. T.; Baldwin, A. J.; Marklund, E. G.; Hochberg, G. K. A.; Benesch, J. L. P.; Robinson, C. V. Bayesian Deconvolution of Mass and Ion Mobility Spectra: From Binary Interactions to Polydisperse Ensembles. *Anal. Chem.* **2015**, *87* (8), 4370–4376. https://doi.org/10.1021/acs.analchem.5b00140.

(9) Shevchenko, A.; Tomas, H.; Havliš, J.; Olsen, J. V.; Mann, M. In-Gel Digestion for Mass Spectrometric Characterization of Proteins and Proteomes. *Nat. Protoc.* **2007**. https://doi.org/10.1038/nprot.2006.468.

(10) Wu, D.; Struwe, W. B.; Harvey, D. J.; Ferguson, M. A. J.; Robinson, C. V. N-Glycan Microheterogeneity Regulates Interactions of Plasma Proteins. *Proc. Natl. Acad. Sci. U. S. A.* **2018**. https://doi.org/10.1073/pnas.1807439115.

(11) Tyanova, S.; Temu, T.; Cox, J. The MaxQuant Computational Platform for Mass Spectrometry-Based Shotgun Proteomics. *Nat. Protoc.* **2016**. https://doi.org/10.1038/nprot.2016.136.

(12) Whitmore, L.; Wallace, B. A. Protein Secondary Structure Analyses from Circular Dichroism Spectroscopy: Methods and Reference Databases. *Biopolymers* **2008**, *89* (5), 392–400. https://doi.org/10.1002/bip.20853.

(13) Johnson, W. C. Analysing Protein Circular Dichroism Spectra for Accurate Secondary Structures". *Proteins Struct. Funct. Genet.* **1999**, *35* (January), 307–312.

(14) Abdul-Gader, A.; Miles, A. J.; Wallace, B. A. A Reference Dataset for the Analyses of Membrane Protein Secondary Structures and Transmembrane Residuesusing Circular Dichroism Spectroscopy. *Bioinformatics* **2011**, *27* (12), 1630–1636. https://doi.org/10.1093/bioinformatics/btr234.
